# Urinary and faecal N-methylhistamine concentrations do not serve as markers for mast cell activation or clinical disease activity in dogs with chronic enteropathies

**DOI:** 10.1186/s13028-014-0090-y

**Published:** 2014-12-21

**Authors:** Kristin P Anfinsen, Nora Berghoff, Simon L Priestnall, Jan S Suchodolski, Jörg M Steiner, Karin Allenspach

**Affiliations:** Department of Veterinary Clinical Sciences, Royal Veterinary College, University of London, Hatfield, AL9 7TA England; Department of Pathology & Pathogen Biology, Royal Veterinary College, University of London, Hatfield, AL9 7TA England; Gastrointestinal Laboratory, Department of Small Animal Clinical Sciences, College of Veterinary Medicine, Texas A&M University, College Station, TX 77843-4474 USA; Current address: NMBU School of Veterinary Science, Department of Companion Animal Clinical Sciences, Norwegian University of Life Sciences (NMBU), Box 8146 Dep., N-0033 Oslo, Norway

**Keywords:** N-methylhistamine, Mast cell, Canine chronic enteropathy

## Abstract

**Background:**

This study sought to correlate faecal and urinary N-methylhistamine (NMH) concentrations with resting versus degranulated duodenal mast cell numbers in dogs with chronic enteropathies (CE), and investigate correlations between intestinal mast cell activation and clinical severity of disease as assessed by canine chronic enteropathy clinical activity index (CCECAI), and between urinary and faecal NMH concentrations, mast cell numbers, and histopathological scores. Twenty-eight dogs with CE were included. Duodenal biopsies were stained with haematoxylin and eosin (H&E), toluidine blue, and by immunohistochemical labelling for tryptase. Duodenal biopsies were assigned a histopathological severity score, and duodenal mast cell numbers were counted in five high-power fields after metachromatic and immunohistochemical staining. Faecal and urinary NMH concentrations were measured by gas chromatography–mass spectrometry.

**Results:**

There was no correlation between the CCECAI and faecal or urinary NMH concentrations, mast cell numbers, or histopathological score – or between faecal or urinary NMH concentration and mast cell numbers. Post hoc analysis revealed a statistically significant difference in toluidine blue positive mast cells between two treatment groups (exclusion diet with/without metronidazole versus immunosuppression (IS)), with higher numbers among dogs not requiring IS.

**Conclusion:**

Faecal and urinary NMH concentrations and duodenal mast cell numbers were not useful indicators of severity of disease as assessed by the CCECAI or histological evaluation. The number of duodenal mast cells was higher in dogs that did not need IS, i.e. in dogs responding to an exclusion diet (with/without metronidazole), than in dogs requiring IS. Further studies comparing the role of mast cells in dogs with different forms of CE are needed.

## Background

Canine chronic enteropathies (CE) are characterised by inflammatory cell infiltration of the intestinal lamina propria, the most common of which is lymphoplasmacytic [1,2]. This condition can retrospectively be classified into food-responsive disease (FRD), antibiotic-responsive disease (ARD), and idiopathic inflammatory bowel disease (IBD) according to response to treatment [1,3,4]. Clinical markers of disease are sparse to date; few parameters have been found to predict the need for immunosuppression (IS) versus diet or antimicrobial treatment alone, or to predict refractoriness to treatment and hence prognosis [2,5].

Aberrant immune responses against antigens such as the intestinal microbiota or food allergens in the intestinal lumen are thought to play a role in the development of canine CE [3,6,7]. These aberrant responses may in part be attributable to a type I hypersensitivity reaction, which involves degranulation of mast cells in the intestinal mucosa [8]. Moreover, mast cells have been suggested to play a role in the pathogenesis of canine and human enteropathies and may be a marker of disease severity [8-12]. Studies investigating numbers of mast cells in human IBD (i.e. Crohn’s disease (CD) and ulcerative colitis (UC)) have found conflicting results [13-16]; possibly reflecting the importance of mast cell activation rather than absolute numbers in the mucosa [12].

German and colleagues investigated leukocyte subsets in the canine intestine based on biopsies obtained from 10 dogs of various breeds with no known enteropathies, providing a reference for future studies on mast cell numbers in the canine intestinal mucosa [17]. A few years later, the same investigators reported leukocyte subsets in the duodenal mucosa of 27 dogs with chronic gastrointestinal disease [18]. In the latter study, a lower number of mast cells was detected in 9 dogs with idiopathic IBD (excluding FRD and ARD) compared to 11 dogs with ARD and 23 healthy controls. This was in contrast to Locher *et al*. [9], who reported a higher number of mast cells in the stomach and duodenum in 20 dogs with IBD compared to 9 healthy controls.

Mast cells are bone marrow-derived cells reaching maturity within the tissues, with characteristic metachromatically staining granules containing inflammatory mediators such as heparin and histamine [19]. Cross-linking of IgE molecules on the surface of mast cells leads to degranulation and release of the inflammatory mediators, evoking inflammatory changes. Once mast cells have released their granules, their ability to stain in a metachromatic fashion diminishes. The metachromatic properties of mast cell granules are attributed to heparin, accounting for the difficulties identifying mast cells that have released the contents of their granules. However, monoclonal antibodies for tryptase and chymase are able to identify small amounts of these mast cell-specific proteases, providing a method for detecting even degranulated (i.e. activated) mast cells [12]. The content of tryptase versus chymase in mast cells varies between tissue localisation [20]. In the human and canine gastrointestinal tract, mast cells that only contain tryptase (MC_T_) have been shown to outnumber cells containing both tryptase and chymase (MC_TC_), or cells only containing chymase (MC_C_) [9,10,20,21]. These findings suggest that monoclonal antibodies for tryptase most accurately identify activated intestinal mast cells.

As the quantification of intestinal mast cell numbers is difficult, serum markers for the detection of mediators released from mast cells have been described. Histamine is one of the major inflammatory mediators released from activated mast cells, and histamine concentrations may directly reflect the degree of mast cell activation [22]. However, histamine in serum is rapidly metabolised to form N-methylhistamine (NMH) and imidazole acetaldehyde [23]. Due to the short half-life of serum histamine [24], measurement of metabolites in body excretions (urine and faeces) is considered to more accurately reflect the overall mast cell activity [23]. Moreover, NMH is an easily measurable parameter that can be analysed in specimens obtained by non-invasive means, i.e., in faeces or voided urine [25]. A gas chromatography–mass spectrometry (GC-MS) method for measurement of NMH concentrations in canine urine samples and faecal extracts has recently been validated [25]. There is increasing evidence that NMH concentration is elevated in the urine and faeces of some dogs with gastrointestinal disease, such as racing sled dogs with gastro-intestinal ulcerations [26], Lundehunds with protein-losing enteropathy [27], and in dogs with CE [28]. Further, it has been shown that NMH concentrations are increased in plasma and urine of humans with IBD – with higher concentrations associated with a higher clinical activity index, and decreasing concentrations corresponding to clinical remission following therapy [29,30]. Only one study has so far investigated a possible correlation between severity of clinical disease and histamine concentrations in canine CE [28]. An increase in faecal NMH was found in dogs with chronic gastrointestinal disease compared to healthy controls, and one quarter of the dogs with CE had increased urinary NMH. However, concentrations did not correlate with severity of disease as assessed by the canine chronic enteropathy clinical activity index (CCECAI) [5].

The aim of the present study was to correlate faecal and urinary NMH concentrations with resting (i.e., metachromatically staining) versus degranulated (i.e., identified by monoclonal tryptase antibodies) duodenal mast cell numbers, hypothesising that urinary and/or faecal NMH concentrations would increase with increasing mast cell degranulation and hence serve as markers for mast cell activity. We further sought to investigate possible correlations between mast cell activation (assessed by degranulation and NMH concentrations) and clinical disease activity index (CCECAI), hypothesising that we would find evidence of increased mast cell activity in patients with clinically more severe disease. Finally, we investigated the relationship between both NMH concentrations and mast cell numbers versus histopathological scores, the latter assessed according to the standards defined by the World Small Animal Veterinary Association (WSAVA) Gastrointestinal Standardization Group [31].

## Materials and methods

### Selection of cases

The archived serum and histology samples for this study have been taken for diagnostic purposes only and are residual samples already stored and available in the Royal Veterinary College (RVC) archive. They were originally obtained with informed owner consent under the Veterinary Surgeons Act (residual clause) and approved by the RVC Ethics and Welfare Committee. The Clinical Investigations Centre at the RVC has archived samples from dogs with CE since 2005; consisting of serum, plasma, urine, faeces, gastrointestinal biopsies, or any combination thereof. An identification number labels all samples, allowing retrospective review of clinical data from the Queen Mother Hospital for Animals’ electronic and/or paper records. The enteropathy archive was searched for dogs with a final diagnosis of CE for which urine, faeces, duodenal biopsies, and clinical data were available. Previously reported groups of clinically healthy dogs were used as controls for establishing control ranges for urinary and faecal NMH concentrations (n = 6 and n = 49, respectively) [25,28].

### Clinical diagnosis and laboratory methods

All dogs selected for the study had presented to the Queen Mother Hospital for Animals (QMHA) with a chronic (3 weeks or more) history of vomiting and/or diarrhoea. Exclusion of extra gastrointestinal disease as a cause of the presenting signs was based on results from routine blood work (haematology and serum biochemistry), urinalysis, faecal parasitology, endocrine tests (basal cortisol or ACTH stimulation test), serum trypsine-like immunoreactivity, and abdominal ultrasound examination. Gastroduodenoscopy was performed in all dogs, and duodenal biopsies were fixed in phosphate-buffered formalin solution followed by paraffin embedding. Clinical disease activity index (expressed as CCECAI) was assigned to each dog at the time of presentation (n = 6) or retrospectively by review of paper and electronic records (n = 22) by one of the investigators (KPA) [5]. All clients seen at the QMHA are asked questions based on a standardised questionnaire, facilitating retrospective determination of the CCECAI. Treatments were initiated stepwise according to clinician preference at the time, and were recorded as exclusion diet (i.e. novel or hydrolysed protein diet fed exclusively), antimicrobial therapy (metronidazole), and/or immunosuppressive therapy (prednisolone, azathioprine, and/or cyclosporine).

Duodenal biopsies were stained with haematoxylin and eosin (H&E) for routine histopathological evaluation – all of which were examined by board-certified pathologists at the time of the initial investigation. A single board-certified pathologist (SP), blinded to all other information about the dogs, reassessed the duodenal biopsies to provide a histopathological score according to the standards set by the WSAVA Gastrointestinal Standardization Group [31].

**Figure 1:**
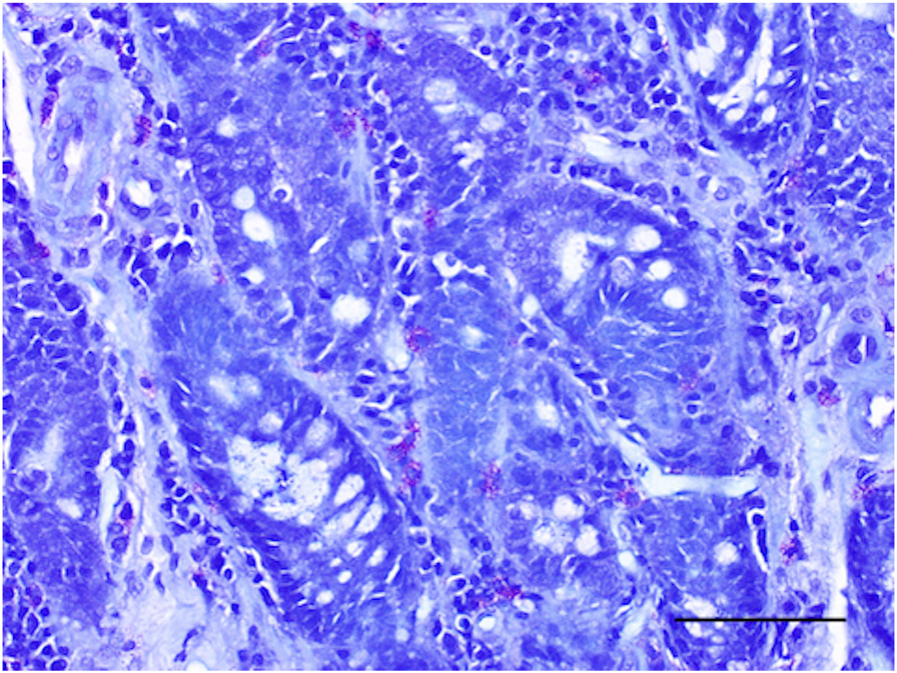
**Duodenal section from a dog with chronic enteropathy stained with toluidine blue for metachromatic staining of mast cells (×200).** Purple cells represent mast cells. Bar = 100 μm.

**Figure 2:**
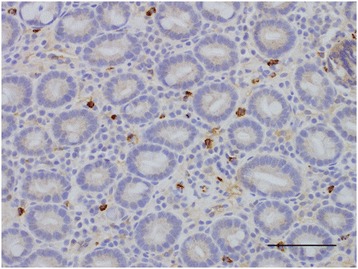
**Duodenal section from a dog with chronic enteropathy labelled immunohistochemically with anti-human mast cell tryptase (×200).** Brown cells represent mast cells. Bar = 100 μm.

Metachromatic staining with toluidine blue was performed as described elsewhere [12] (Figure 1). Immunohistochemical staining for mast cell tryptase was performed using an automated staining machine (Leica BondMax) and amplification kit (Leica Refine), according to the standard immunohistochemical protocol used at the RVC Diagnostic Laboratory (Figure 2). Briefly, 4 μm sections of duodenal mucosa were pretreated with a pH 9.0 retrieval solution (Dako Epitope Retrieval 2 solution, Dako, Ely) for 10 minutes then a mouse anti-human mast cell tryptase monoclonal antibody (Dako) was used at a 1:800 dilution. Sections were counterstained with haematoxylin.

Numbers of mucosal mast cells were assessed in duodenal biopsies by metachromatic staining (toluidine blue; MC_TB_) and by immunohistochemical labelling for tryptase (MC_T_) [21]. Duodenal mast cell numbers were counted in five high-power fields (×40) for each dog and each staining method by one of the authors (KPA).

Faecal and urine samples (either voided or collected by cystocentesis) from all dogs were frozen at −80°C, for up to 2.5 years, until further analysis. Faecal and urinary NMH concentrations were determined by GC-MS as described elsewhere [25,32]. The assay has previously been analytically validated for use in dogs at the Texas A&M Gastroenterology Laboratory [25].

### Statistical analyses

Spearman’s rank correlation coefficient was calculated to assess correlation between faecal and urinary NMH concentrations with clinical severity of disease (expressed as CCECAI) and histological severity of disease (expressed as WSAVA score), and between mast cell numbers and clinical and histological severity of disease. Normality was assessed by the Shapiro Wilk test. For non-normally distributed data, Mann–Whitney U test was used for comparison of mast cell numbers and NMH concentrations between treatment groups (diet and/or antimicrobials versus immunosuppressive treatment). T-test was used for normally distributed data. Statistical significance level was set at *P* < 0.05.

## Results

Between November 2005 and August 2011, samples from 259 dogs with a recorded diagnosis consistent with CE (e.g. IBD, enteropathy, protein-losing enteropathy, food-responsive enteropathy, gastroenteritis) were archived. For 32 of these dogs, urine, faeces, and duodenal biopsy samples were recorded to be available. Retrospective review of records from these dogs led to exclusion of one dog due to a final diagnosis of histiocytic ulcerative colitis. Another 3 dogs were excluded due to concurrent severe extra-intestinal disease, with potentially overlapping clinical signs (renal lymphoma, hepatic lymphoma, and immune-mediated haemolytic anaemia). The final study population consisted of 28 dogs, 21 of which had all samples (duodenal biopsies, urine and faeces) present in the archive. (Duodenal and faecal samples were available from 24 dogs each; from 1 dog both faecal and duodenal samples were missing).

Median age of the dogs in the study population was 4 years (range: 13 months-10 years); 13 females (10 spayed) and 15 males (9 castrated). Eight breeds were represented, including 8 Labrador retrievers, 3 German shepherd dogs, 2 Cocker spaniels, 2 Cross breed dogs, and one each of: Cavalier King Charles spaniel, Siberian Husky, Keeshond, Staffordshire Bull terrier, Jack Russell terrier, Yorkshire terrier, Greyhound, Newfoundland, Weimaraner, Utagon, Dogue de Bordeaux, Golden retriever, and Miniature schnauzer.

Median urinary NMH concentration was 97 ng/mg creatinine (range 5–249 ng/mg creatinine), and median faecal NMH concentration was 48 ng/g (range 0–1,451 ng/g) (Table 1). These concentrations were within the normal range (0–136 ng/mg creatinine for urinary NMH and 0–191 ng/g for faecal NMH) [25,28], and not significantly different from the 3-day mean faecal NMH concentrations previously reported in 49 healthy control dogs (53 ng/g, range 9–252 ng/g) [28]. Six dogs had urinary NMH concentrations above the upper limit of the reference interval, and 4 dogs had faecal NMH higher than the upper limit of the reference interval; 2 dogs had both urinary and faecal NMH concentrations above the upper limit of the reference interval. Median CCECAI was 6 (range 2–13), and median histopathology score 2 (range 0–9). In 2 dogs (with histopathology scores of 0 and 4), the duodenal biopsies were of inadequate quality as defined by the WSAVA Gastrointestinal Standardization group, and these biopsies were excluded from further analyses of histopathological severity.

**Table 1:** **Summary of clinical and clinicopathological data in 28 dogs with chronic enteropathy**

**Dog**	**Age (years)**	**Sex**	**CCECAI**	**Therapy**	**Fecal NMH**	**Urinary NMH**	**Mast cells (mean number/10 hpf)**		**Histopathology duodenum**	
					**≤191(ng/g)**	**≤136(ng/mg creatinine)**	**TB**	**IHC**	**WSAVA score**	**Infiltrate**
1	9y 4 m	FN	12	D, IS	74	176	16.6	31.4	3	LP
2	1y 5 m	MN	6	D, M	-	46	3.2	21	0	N/A
3	3y 8 m	MN	5	D, M	40	86	22.6	22.6	1	LP
4	2y 1 m	F	7	D, M	0	32	2	16	1	neut
5	1y 3 m	M	5	D	-	474	1.4	10	0	N/A
6	10y	MN	9	D, M	-	69	11.6	16.4	3	LP
7	1y 6 m	FN	6	D, IS	165	108	1.2	2.6	0	N/A
8	7y 9 m	FN	7	D, M, IS	789	186	2.6	13.4	2	-
9	5y 8 m	MN	9	D	57	132	9.2	28	0	N/A
10	3y 1 m	MN	3	D, M	139	95	10	11.8	1	-
11	6y 5 m	MN	4	D	1451	62	6.4	10.2	5	LP, eos
12	2y 7 m	MN	6	D, M	0	249	8	27.4	5	eos
13	4y 11 m	FN	7	D, IS	54	112	2.2	2	1	-
14	8y	M	13	D, M	272	114	3.8	9.8	4	LP
15	2y 6 m	M	7	D, M	98	68	1.6	12.2	5	LP*
16	4y	F	8	D, IS	1150	163	0.2	8.6	5	eos
17	4y 4 m	M	9	D, IS	29	19	1	4.2	1	-
18	3y 10 m	FN	6	D	0	53	0.4	7.8	2	LP
19	1y 1 m	MN	5	D	0	100	8.8	4.2	9	LP, eos**
20	1y 2 m	MN	5	D	0	99	3.6	9.2	1	-
21	6y 2 m	FN	9	D, IS	0	43	0.1	1.2	3	LP
22	3y 3 m	FN	2	D	0	46	0.4	2.2	3	LP*
23	6y 6 m	M	6	D	131	53	2.8	10.4	6	LP
24	8y 2 m	FN	9	D	105	5	9.2	6.6	0	N/A
25	4y 5 m	M	11	D, IS	41	76	-	-	-	-
26	1y 1 m	F	6	D, M, IS	-	52	-	-	-	-
27	8y 7 m	FN	4	IS	0	116	-	-	-	-
28	4y	FN	6	D, M	38	151	-	-	-	-
Median			6		47.5	90.5	3	10.1	2	
Min			2		0	5	0.1	1.2	0	
Max			13		1451	474	22.6	31.4	9	

CCECAI, canine chronic enteropathy clinical activity index; NMH, N-methylhistamine; hpf, high-power field; TB, toluidine blue; IHC, immunohistochemistry (tryptase); WSAVA, World Small Animal Veterinary Association; FN, female neutered; MN, male neutered; D, diet (novel or hydrolysed protein); M, metronidazole; IS, immunosuppressive drug (prednisolone, azathioprine, cyclosporine); LP, lymphoplasmacytic; neut, neutrophilic; eos, eosinophilic; *, moderate; **, marked; N/A, not applicable; − data missing.

There was no correlation between the CCECAI and faecal or urinary NMH concentrations (Spearman’s rho 0.23 and −0.032, respectively), mast cell numbers (MC_TB_ or MC_T_; rho −0.004 and 0.04, respectively), or histopathology score (rho −0.103). Furthermore, there was no correlation between faecal (rho 0.05 and −0.001 for MC_TB_ and MC_T_, respectively) or urinary (rho 0.18 and 0.26 for MC_TB_ and MC_T_, respectively) NMH concentration and mast cell numbers, nor was there any correlation between urinary and faecal NMH concentrations (rho 0.22).

**Figure 3:**
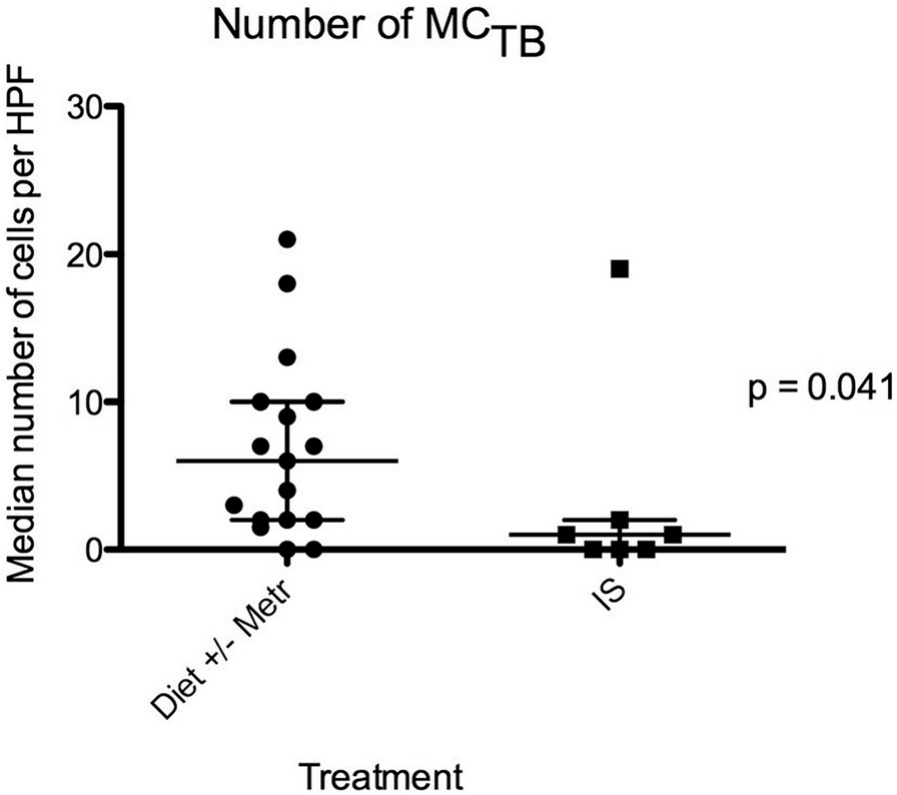
**Median numbers of MC**_**TB**_ **in 5 high power fields (×40) for 24 dogs with chronic enteropathy, grouped based on treatment.** IS = treated by immunosuppression; Metr = treated with metronidazole. Lines represent median and interquartile range.

**Figure 4:**
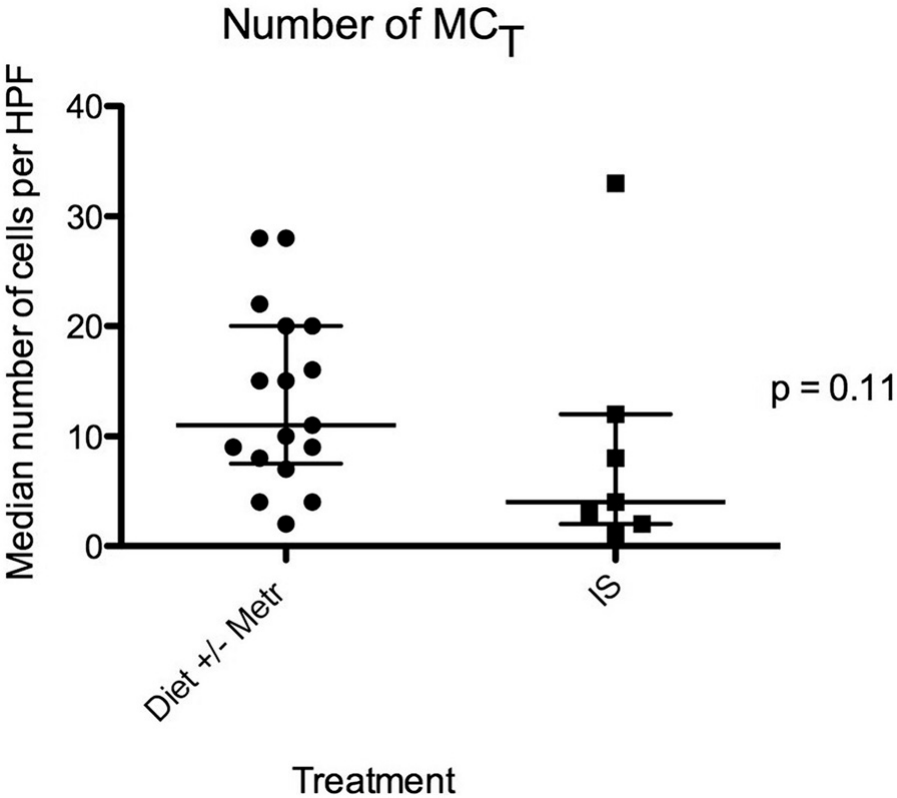
**Median numbers of MC**_**T**_ **in 5 high power fields (×40) for 24 dogs with chronic enteropathy, grouped based on treatment.** IS = treated by immunosuppression; Metr = treated with metronidazole. Lines represent median and interquartile range.

**Figure 5:**
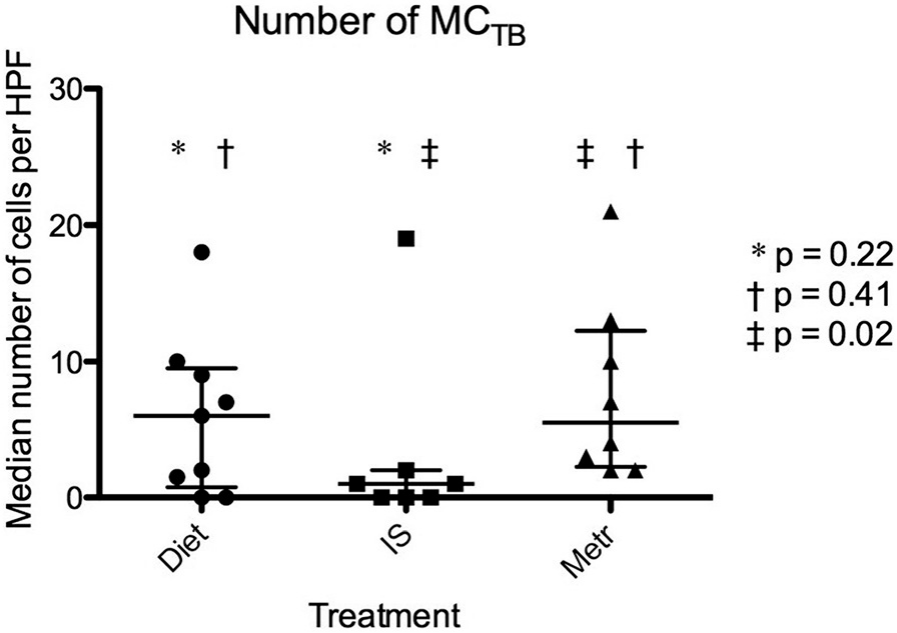
**Median numbers of MC**_**TB**_ **in 5 high power fields (×40) for 24 dogs with chronic enteropathy, separated by treatment group.** IS = treated by immunosuppression; Metr = treated with metronidazole. Lines represent median and interquartile range.

Treatment initiated or advised by clinicians at the QMHA was recorded for each dog, and consisted of an exclusion diet with (n = 9) or without (n = 9) metronidazole, or IS (n = 10; these dogs had failed treatment trials with diet and metronidazole, either prior to or after referral to the QMHA). Biopsies from 4 of the dogs (3 treated with IS, one with diet and metronidazole) were missing from the archive. Due to the low number of dogs, assessment of differences between treatment groups was not attempted in this study. However, the data gave an impression of higher numbers of mast cells detected in duodenal biopsies from dogs in which IS therapy was not necessary; i.e. dogs responding to an exclusion diet with or without metronidazole. Post hoc analysis revealed that dogs eventually requiring immunosuppressive therapy had less MC_TB_ (*P* = 0.041) in their pre-treatment biopsies than those responding to an exclusion diet, with or without metronidazole, (Figure 3). There was no statistically significant difference between these groups for numbers of MC_T_ (*P* = 0.105; Figure 4), and there was no difference between numbers of MC_TB_ in dogs treated with an exclusion diet and metronidazole versus an exclusion diet alone (*P* = 0.41; Figure 5). Faecal and urinary NMH concentrations did not differ between treatment groups (*P* = 0.52 and *P* = 0.95, respectively).

## Discussion

We were unable to find a correlation between intestinal mast cell numbers and clinical severity of disease as assessed by CCECAI based on the dogs enrolled in this study. Previous studies assessing mast cell numbers in dogs with various enteropathies have yielded conflicting results, which may be partially explained by differences in fixation and/or staining techniques [3,33]. More specifically, it has been postulated that mast cell degranulation with release of inflammatory mediators could explain a decrease in detectable mast cells in diseased tissue, as degranulated mast cells stain poorly [3,12]. Immunolabelling for mast cell proteases (i.e. tryptase and/or chymase) enables identification of degranulated cells. A decrease in metachromatically staining mast cells in combination with a constant to increasing number of tryptase positive mast cells in patients with inflammatory intestinal disease would support a role of mast cell mediators in that particular patient. Therefore, we investigated the number of both metachromatically staining mast cells (MC_TB_) and of MC_T_. The lack of correlation between either of these and CCECAI could suggest that mast cells and mast cell mediators are not major contributors to the clinical signs observed in canine patients with CE.

Studies investigating the number of mast cells in human patients with IBD (i.e. CD and UC) have also reported conflicting results. Dvorak *et al.* [13] identified mast cells by electron microscopy and found increased numbers of mast cells in the ileal mucosa of patients with CD compared to healthy controls. Nishida *et al*. [15] utilised immunohistochemical staining for tryptase and described higher mast cell numbers in patients with both CD and UC than in healthy controls. In contrast, Nolte *et al.* [34] reported increased numbers of mast cells in patients with UC, but similar numbers in those with CD, when comparing metachromatically staining mast cells in humans with IBD to healthy controls. Mast cell numbers were higher in inflamed tissue than normal tissue from both patient groups. Balász *et al.* [14] also reported higher numbers of metachromatically staining mast cells in patients with active UC than in patients in remission. In contrast, King *et al.* [16] found a decreased number of mast cells in areas of active inflammation in patients with UC compared to normal colonic mucosa from the same individuals.

The discrepancies in both human and canine studies may partially be explained by different staining techniques, resulting in a variable ability to identify intact versus degranulated mast cells [35]. More specifically, mast cell degranulation could explain why German *et al.* [18] found decreased numbers of mast cells identified by toluidine blue in dogs with IBD, whereas Locher et al. showed numbers of duodenal tryptase positive mast cells to be increased in dogs with this condition [3,9]. Bearing this in mind, Kleinschmidt *et al.* [10] sought to identify the number of metachromatically staining mast cells (using kresylecht-violet; MC_KEV_) versus tryptase and/or chymase positive mast cells (MC_total_) in dogs with inflammatory enteropathies versus controls. Although their findings generally showed a decrease in mast cell numbers in diseased dogs, they identified higher numbers of MC_total_ versus MC_KEV_ in 14 of 19 small intestinal samples from inflamed areas in affected dogs, whereas this was the case for only 8 of 20 unaffected areas of the small intestine from these patients, suggesting an association between mast cell activation and intestinal inflammation in this population of dogs.

Post hoc analysis of the data in this study revealed higher numbers of MC_TB_ in dogs treated with an exclusion diet (with or without antimicrobial treatment) versus dogs requiring IS to control clinical signs (*P* = 0.041), supporting a role of mast cell infiltration and activation in this subset of dogs with CE. The low number of cases can most likely explain the lack of a statistically significant difference between numbers of MC_T_. However, it could also suggest increased mast cell activation (leading to lower numbers of MC_TB_, but not MC_T_) in dogs requiring IS. Interestingly, German et al. reported similar findings in 27 dogs with CE [18]. Dogs classified as having IBD (i.e. requiring IS) had significantly lower numbers of MC_TB_ than dogs with ARD, and dogs with FRD appeared to have similar numbers of MC_TB_ as the dogs with ARD; lack of a significant difference between dogs in the dogs with FRD and IBD in our study could be due to a low number of dogs with FRD (n = 6). Lower amounts of IgE antibodies detected in the faeces of Soft Coated Wheaten Terriers fed an exclusion diet, and peak faecal IgE levels measured following feeding with a provocative diet, lends some support to the theory that mast cell activation may be involved in the pathogenesis of FRD [36].

Using similar techniques as the present study for metachromatic staining (toluidine blue) and counting of duodenal mucosal mast cell numbers, Berghoff *et al.* [28] reported a median mast cell count of 4.4 per high-power field (range 0–17, n = 11) in dogs with CE. These numbers are similar to ours, and in line with our findings, the degree of mast cell infiltration did not correlate with the urinary or faecal NMH. Their study did not include information about treatment, precluding any such comparisons.

In accordance with previous studies, we did not find any correlation between CCECAI and the histopathological score – underlining the need for additional markers of clinical disease and response to treatment [37,38]. In human patients with IBD (CD or UC), urinary NMH has been correlated to clinical disease activity and severity of lesions observed by endoscopy, with increased urinary NMH concentrations corresponding to active disease, and urinary NMH concentrations during clinical remission being similar to those of healthy controls [29,30]. In our study, neither urinary nor faecal concentrations of NMH correlated with clinical disease activity (CCECAI), and in contrary to mast cell numbers, there was no difference in urinary or faecal NMH concentrations between treatment groups. This could again reflect low numbers of cases. Moreover, it is likely that mast cell activation and release of histamine play a role only in a subgroup of dogs, further decreasing the power of our study, as we included dogs with ARD, FRD, and IBD. Finally, we cannot exclude inaccurate results due to decay of urinary and faecal NMH during storage, although there was no indication of lower values found in the older compared to the newer samples (data not shown).

Previous studies have suggested a role of histamine release in select groups of dogs with enteropathy. Vaden *et al.* [39] reported decreased release of histamine from jejunal mast cells following *in vitro* stimulation of biopsies from Soft Coated Wheaten Terriers with protein-losing enteropathy and/or –nephropathy, and postulated that this could reflect depletion of histamine due to ongoing degranulation. Berghoff *et al.* [27] found increased faecal NMH concentrations in Norwegian Lundehunds with chronic gastrointestinal disease compared to healthy controls and also in racing Alaskan sled-dogs, prone to gastrointestinal ulceration, compared to working Retrievers [26]. These findings were followed up by the first study investigating a potential correlation between faecal and urinary NMH concentrations and clinical severity of disease in dogs with CE [28]. In line with our findings, there was no correlation between faecal and urinary NMH, or between either faecal or urinary NMH concentrations and the CCECAI scores. However, dogs with CE had higher mean concentrations of faecal NMH than healthy control dogs, and 4/16 dogs had increased urinary and faecal NMH concentrations (only 1 dog had an increase in both). The authors suggested that histamine release was likely to play a role in some dogs with CE. Interestingly, the 4 dogs with increased urinary NMH concentrations all had moderate inflammatory infiltrate in the duodenal mucosa, whereas the inflammatory changes in the 12 dogs with normal urinary NMH were moderate in one and mild in the remaining dogs. These findings contrast ours, as none of the dogs with moderate or marked duodenal mucosal inflammation (dog number 15, 19, 22) had increased urinary NMH. This discrepancy could be due to the low number of cases in both studies, and in particular, the generally mild histopathological changes in our population. The proportion of dogs with increased faecal or urinary NMH in our study (17 and 21%, respectively) was comparable to the 25% reported by Berghoff *et al.* [28].

In our study, incorporating a histopathology score and mast cell numbers unfortunately did not help to elucidate the potential clinical utility of measuring NMH, as neither faecal nor urinary NMH concentrations correlated with mast cell numbers or histopathological severity. However, it did lend some support to mast cell infiltration playing a role in a subgroup of dogs with CE – revealing a higher number of mast cells by metachromatic staining (MC_TB_) in dogs with FRD and ARD. As there was no significant difference in numbers of MC_T_, increased degranulation in dogs with IBD could not be ruled out, but this finding was thought to more likely be due to low numbers and hence lack of power to detect a difference in this group.

Another limitation of this study, was that only endoscopically obtained duodenal biopsies were available. It is possible that a correlation between NMH concentrations and clinical severity (CCECAI) or mast cell numbers could have been detected if jejunal, ileal and/or full-thickness biopsies had been available for analyses. Recent studies have shown histological discrepancies between duodenal and ileal samples from dogs with CE, and indicated a higher frequency of ileal versus duodenal lesions – although the number of mast cells were not assessed in these studies [38,40]. Kleinschmidt *et al.* [10] utilised full-thickness biopsies from all segments of the canine gastrointestinal tract to assess intestinal mast cell numbers and distribution. These investigators described lower mast cell numbers in the submucosa than the lamina propria, rendering the absence of deeper layers (i.e. the submucosa and muscularis) in the biopsies an unlikely cause of our negative findings. However, in dogs with lymphoplasmacytic enteritis, lower numbers of MC_T_ were reported in the duodenum and jejunum compared to the ileum. Including assessment of ileal biopsies would have strengthened the present study, but was not feasible due to its retrospective nature.

## Conclusions

Our study did not show any correlations between urinary or faecal NMH concentrations and clinical disease severity or numbers of intestinal mast cells. However, there was a trend of increased numbers of intestinal mast cells in dogs responding to diet and/or antimicrobial therapy compared to those requiring IS. Further studies designed to look at differences in intestinal mast cells between dogs with the three major forms of CE (FRD, ARD, and idiopathic IBD) are required to investigate the significance of this observation.

## Abbreviations

ARD: Antibiotic-responsive disease
CCECAI: Canine chronic enteropathy clinical activity index
CD: Crohn’s disease
CE: Chronic enteropathy
FRD: Food-responsive disease
GC-MS: Gas chromatography–mass spectrometry
IBD: Inflammatory bowel disease
IS: Immunosuppression
MC_C_: Mast cells containing only chymase
MC_KEV_: Mast cells stained metachromatically using kresylecht-violet
MC_T_: Mast cells containing only tryptase
MC_TB_: Mast cells stained metachromatically using toluidine blue
MC_TC_: Mast cells containing both tryptase and chymase
MC_total_: Mast cells containing tryptase and/or chymase
NMH: N-methylhistamine
UC: Ulcerative colitis
WSAVA: World Small Animal Veterinary Association
